# Multi-parametric [^18^F]PI-2620 tau PET/MRI for the phenotyping of different Alzheimer’s disease variants

**DOI:** 10.1007/s00259-025-07135-z

**Published:** 2025-02-12

**Authors:** Michael Rullmann, Dylan Henssen, Juliana T. Melasch, Cordula Scherlach, Dorothee Saur, Matthias L. Schroeter, Solveig Tiepolt, Norman Koglin, Andrew W. Stephens, Swen Hesse, Maria Strauss, Matthias Brendel, Olena Mishchenko, Andreas Schildan, Joseph Classen, Karl-Titus Hoffmann, Osama Sabri, Henryk Barthel, for the German Imaging Initiative for Tauopathies (GII4T)

**Affiliations:** 1 Department of Nuclear Medicine, University of Leipzig Medical Center Leipzig, Leipzig, Germany; 2 Department of Medical Imaging, Radboud University Medical Center, Nijmegen, The Netherlands; 3 Department of Neuroradiology, University of Leipzig Medical Center Leipzig, Leipzig, Germany; 4 Department of Neurology, University of Leipzig Medical Center Leipzig, Leipzig, Germany; 5 Clinic for Cognitive Neurology, University of Leipzig Medical Center Leipzig, Leipzig, Germany; 6 Max Planck Institute for Human Cognitive and Brain Sciences, Leipzig, Germany; 7 Life Molecular Imaging, Berlin, Germany; 8 Department of Psychiatry, University of Leipzig Medical Center Leipzig, Leipzig, Germany; 9 Department of Nuclear Medicine, LMU University Hospital, LMU Munich, Munich, Germany; 10 Munich Cluster for Systems Neurology (SyNergy), Munich, Germany; 11 German Center for Neurodegenerative Diseases (DZNE), Munich, Germany; 12 Department of Nuclear Medicine, Hospital Dessau, Dessau, Germany

**Keywords:** Tau PET, MRI, fMRI, DTI, Neuroimaging, Alzheimer’s disease, Alzheimer’s disease variants

## Abstract

**Purpose:**

Heterogeneity in clinical phenotypes has led to the description of different phenotypes of Alzheimer’s disease (AD). Besides the most frequent amnestic variant of AD (aAD), patients presenting with language deficits are diagnosed with logopenic variant primary progressive aphasia (lvPPA), whereas patients presenting with visual deficits are classified as posterior cortical atrophy (PCA).

**Methods:**

This study set out to investigate the value of a multi-parametric [^18^F]PI-2620 tau PET/MRI protocol to distinguish aAD, lvPPA and PCA to support clinical diagnosis in 32 patients. Phenotype-specific information about tau accumulation, relative perfusion, grey matter density, functional network alterations and white matter microstructural alterations was collected.

**Results:**

The aAD patients showed significantly higher tau accumulation, relative hypoperfusion and grey matter density loss in the temporal lobes compared to PCA and lvPPA patients. PCA patients, on the other hand, showed significantly higher tau accumulation in the occipital lobe as compared to aAD patients. Relative hypoperfusion in the occipital lobe and loss of functional connectivity of the posterior cingulate cortex to supplementary visual cortical regions helped to distinguish PCA from lvPPA. Tau accumulation in the cerebellum and microstructural changes in the cingulum were found to help differentiate lvPPA from aAD.

**Conclusion:**

This study highlights structural and functional differences between patients with different AD phenotypes. Differences in regional tau PET signals suggest that refinements in the Braak staging system are needed for the non-aAD cases. These patterns of tau accumulation align with the cascading network failure hypothesis, though more research is needed to warrant the here presented results in larger patient cohorts.

## Introduction

Alzheimer’s disease (AD) is a gradually progressive neurodegenerative disorder which is biologically characterized by the toxic accumulation of misfolded amyloid-β (amyloid plaques) and tau (neurofibrillary tangles) in cortical brain tissue, leading to neurodegeneration. Together, these pathological changes form the fundament of the biological construct that underpins AD known as the ATN model which was proposed by the National Institute on Aging and Alzheimer's Association in 2018 [1]. Prior to the ATN model, AD was diagnosed during lifetime as a syndromal construct [2, 3]. In amnestic AD (aAD), symptoms are diminished memory function, often accompanied by apathy or depressed mood in early stages [4, 5]. Nevertheless, not all patients present with amnestic symptoms and the heterogeneity in patient presentation led to the definitions of various phenotypes of AD. These other AD variants present with specific deficits in cortical functions [3, 6]. For example, some patients present with impaired visual identification of objects, symbols, words or faces (occipitotemporal variant of posterior cortical atrophy; PCA), whereas some suffered from visuospatial dysfunction (biparietal variant of PCA) [7–9]. Other patients present primarily with progressive impairment in single-world retrieval and in sentence-repetition with spared semantic, syntactic and motor speech abilities, leading to the diagnosis of logopenic variant primary progressive aphasia (lvPPA) [10].

Next to differences in clinical presentation, various differences with regard to amyloid-β status, tau status and patterns of neurodegeneration have been reported. In cerebrospinal fluid samples, increased concentration of tau and decreased concentration of amyloid-β are known to reliably reflect dementia diagnosis in aAD and other AD variants [11–13]. Additionally, studies in which amyloid status was investigated by use of positron emission tomography (PET) showed that amyloid-β accumulation also occurs in lvPPA [14, 15] and PCA [16, 17]. Similarly, studies reporting on the use of tau PET imaging showed that the clinical phenotype of AD variants closely matched regional tau burden [18, 19]. However, refinements in the Braak staging system are necessary to incorporate the non-amnestic AD variants [20]. When focusing on neurodegeneration, it is well-known that distinct patterns of hypometabolism on 2-[^18^F]fluoro-2-deoxy-D-glucose ([^18^F]FDG) PET imaging can be observed. These patterns help distinguishing different AD subtypes, although overlapping regions are likewise present (for an overview see [21]).

To incorporate the three dimensions of the ATN model based on imaging biomarkers, patients would need to undergo multiple imaging sessions, which is associated with ethical (e.g., radiation safety) and financial concerns. Hybrid PET/MRI might be able to overcome these concerns. This study was set out to investigate the value of a multi-parametric [^18^F]PI-2620 tau PET/MRI to distinguish aAD and other AD variants. We hypothesized that hybrid PET/MRI with the second-generation tau PET tracer [^18^F]PI-2620 provides phenotype-specific information in a one stop-shop manner about tau accumulation, brain atrophy, brain perfusion, functional network alterations and white matter microstructural alterations which can be used to differentiate between aAD and PCA and lvPPA.

## Materials and methods

### Participants

The ethics committee of Ludwig-Maximilians-University Munich (application numbers 17–569 and 19–022), the medical faculty of the University of Leipzig (EC number 155/15-ff) and the German Federal Office for Radiation Protection (Bundesamt für Strahlenschutz) approved the study protocol. All patients gave written informed consent before the [^18^F]PI-2620 PET/MRI session.

Subjects with a clinical diagnosis of AD (i.e., aAD, lvPPA or PCA) in combination with positive amyloid-ß status (either obtained by PET imaging or CSF sampling) were included in this study. Criteria for exclusion were any known contraindications for MRI.

To assess the severity of cognitive impairment at time of diagnosis, AD patients underwent the Mini Mental State Examination (MMSE) and/or the Montreal Cognitive Assessment (MoCA) during their neuropsychological screening. In case of missing MMSE scores, the MoCA scores were converted into MMSE scores following the method by Fasnacht et al. [22]. PET/MRI findings were compared with those of a normal population constructed out of two imaging cohorts of healthy controls: A previously described cohort of 10 healthy subjects imaged with [^18^F]PI-2620 was included for normalization of the PET data [23]. In addition, a cohort of healthy controls (*n* = 10) that was used for the analysis of the MRI data was collected at our own institute, using the same scanning system and imaging protocol.

### PET image acquisition

Radiosynthesis of [^18^F]PI-2620 has been described in detail previously [24]. All imaging data were acquired on a hybrid PET/MR system (Biograph mMR, Siemens Healthineers, Erlangen, Germany). At the time of intravenous bolus injection of 281 ± 13 MBq [^18^F]PI-2620 dynamic brain PET data were acquired in 3D list-mode over 60 min, and reconstructed into a 256 × 256 matrix (voxel size: 1.00 × 1.00 × 2.03 mm^3^) using the built-in ordered subset expectation maximization algorithm with 8 iterations, 21 subsets and a 3 mm Gaussian filter. For attenuation correction, the vendor-provided HiRes method was employed. This method combines the individual Dixon attenuation correction approach with a bone attenuation template.

### MRI acquisition

All MRI data were acquired simultaneously to the PET data. A T1-weighted three-dimensional, 1mm isotropic, magnetization prepared 2 rapid acquisition gradient echo (MP2RAGE) sequence was used for structural imaging. This is as this sequence is known to provide a superior gray/white matter contrast to the MP-RAGE sequence [25]. For resting state-functional MRI (rs-fMRI), subjects were asked to keep their eyes open and to fixate on an imaginary point without thinking during the entire scanning session (300 acquired echo planar imaging volumes, voxel size 3 × 3 × 4.2 mm^3^, repetition time 2000 ms, echo time 30 ms and slice thickness 3.5 mm). Diffusion-weighted images (DWI) were acquired from 64 axial slices, with a 1.7 mm isotropic voxel size, with 30 diffusion-encoding gradient directions and a b-value of 1000 s/mm^2^. In addition, four volumes without diffusion weighting with a b-value of 0 s/mm^2^ were recorded for offline motion correction.

### PET image processing

Dynamic PET data were motion-corrected and co-registered with the individual MRI image using PMOD (PMOD Technologies LLC, Zurich, Switzerland). Kinetic modeling was performed as described previously [23]. In brief, we applied the Multilinear Reference Tissue Model 2 [26] with cerebellar cortex (excluding the dentate nucleus) as reference region to generate individual DVR (= BP_ND_ + 1) and R1 parametric images. The DVR reflects the ratio of specific tracer binding to a target relative to a reference region and is calculated using the aforementioned formula where BP_ND_ represents the binding potential for the non-displaceable fraction. R1, on the other hand, represents the relative tracer delivery rate to the target region compared to the reference region. While DVR highlights specific receptor or protein density, R1 serves as an indirect marker of regional blood flow or tracer delivery kinetics. The generated DVR and R1 images were spatially normalized based on the computed normalization parameters of the individual MRI data using SPM12 software (Statistical Parametric Mapping; Wellcome Trust Centre for Neuroimaging, London, UK). After this, PET images were smoothed with an 8 mm full-width at half-maximum Gaussian filter.

### VBM processing

Voxel-based morphometry (VBM) was performed on the MP2RAGE MR images using the computational anatomy toolbox (CAT12) in SPM12. The modulated and warped gray matter density (GMD) maps were smoothed with an 8 mm FWHM Gaussian kernel. Individual total intracranial volume is used as a confounding covariate to correct for different brain sizes in the group-level analyses.

### rs-fMRI processing

Regarding rs-fMRI, the first four recorded volumes of each patient were discarded from further analyses to guarantee steady state of blood oxygen level-dependent (BOLD) signals. The remaining rs-fMRI data were pre-processed using SPM12. Within SPM12, images were slice-time corrected, realigned, normalized to the MNI template and finally smoothed using an 8 mm full-width at half-maximum Gaussian filter. Finally, images were bandpass-filtered and detrended using FSL [27]. On the single-subject level, the images were applied to the general linear model, with one regressor representing the scans acquired over time. To compute the functional connectivity of the default mode network region posterior cingulate cortex (PCC), we extracted the first Eigenvariate of the beta values across all voxels within this brain region. The individual PCC time series were added within the same general linear model as an additional non-interacting regressor and used to test for positive correlations (*i.e.*, strengthened connectivity) of the PCC seed region throughout the entire brain, which results in individual statistical maps.

### DWI processing

Diffusion-weighted MR data were processed using the vendor provided tools (Siemens syngo MR E11 software, Siemens, Munich, Germany) in order to generate fractional anisotropy (FA) maps based on the inline calculation of the diffusion tensor.

### Voxel-based statistical analysis

For rs-fMRI, we calculated the mean beta estimates of the dorsal default mode network [28]. For DVR, R1 and FA maps, we used the inverse of the spatial normalization matrix to transform MNI-based atlas to the native space. The spatially normalized and smoothed DVR, R1 and GMD maps were entered into a group-level two-sampled t-test within SPM12 to test for voxel-wise differences between aAD, PCA and lvPPA patients. Significance was detected with a threshold *p* < 0.001 and with a threshold of *p* < 0.005 and minimum cluster size of 30 voxel for rs-fMRI data. In order to correct for disease progression between subjects, the DVR analyses were corrected for Braak stage [29] by adding the highest Braak stage as a covariate to the voxel-based analysis.

### VOI-based statistical analysis

Depending on the considered image modality, we applied different volume of interest (VOI) sets. For DVR maps, we applied a Braak staging atlas [30]. R1 maps were assessed using an AD-specific mask [31]. GMD was determined in the hippocampus. The region was extracted from the Neuromorphometrics atlas (http://Neuromorphometrics.com/) under academic subscription distributed along with CAT12. We corrected all GMD values for the individual total intracranial volume to correct for different brain sizes. For all modalities, we used two-sampled t-test to compare average VOI values between aAD, lvPPA or PCA patients.

Microstructural integrity of large association tracts involved in memory function was compared between aAD patients, lvPPA patients and PCA patients. Furthermore, to test the hypothesis that microstructural integrity in the visual network is significantly more deteriorated in PCA patients as compared to lvPPA patients and aAD patients, VOIs of the optic radiation on each side were obtained from the HCP842-tractography atlas. To test the hypothesis that microstructural integrity in the language network is more affected in lvPPA patients as compared to PCA patients and aAD patients, VOIs of the arcuate fasciculus on each side were obtained from the HCP842-tractography atlas.

A One-Way ANOVA was used to assess the aforementioned hypotheses that microstructural integrity was different between the aforementioned patient groups. The initial level of significance for the VOI-based analysis of the DTI data was set at p < 0.05, and post-hoc Bonferroni correction was carried out to correct for multiple testing.

## Results

**Fig. 1:**
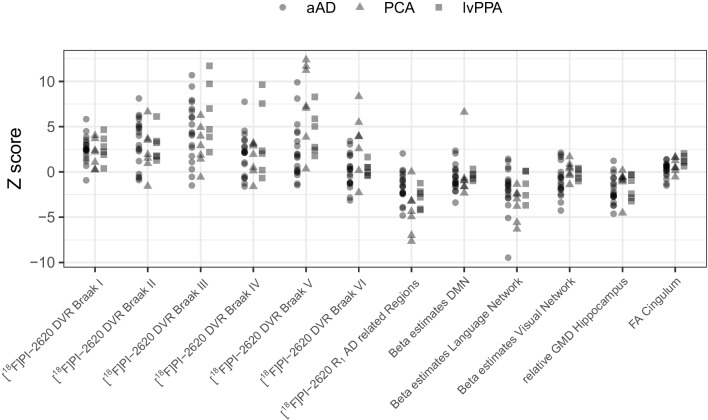
Multi-parametric z-scores as obtained by [^18^F]PI-2620 PET/MRI in different AD subtypes. aAD: amnestic Alzheimer’s disease; DMN: Default mode network; DVR: Distribution volume ratio; FA: Fractional anisotropy; GMD: Grey matter density; lvPPA: logopenic variant primary progressive aphasia; PCA: posterior cortical atrophy; R1: Relative perfusion

In total, 32 patients (mean age: 69.8 ± 9.4 years; 17 females) were included in this study. Nineteen patients suffered from aAD, seven suffered from PCA and six patients suffered from lvPPA. One way ANOVA revealed no significant differences in age (F = 1.101; *p* = 0.346) or MMSE score (F = 2.469; *p* = 0.113) of participants between groups. An overview of the patient’s individual characteristics is provided in Table 1. All patients underwent all imaging procedures, with the exception of one patient who did not wish to undergo the rs-fMRI investigation. Results of the analysis of the multiparametric imaging protocol of aAD patients, lvPPA patients and PCA patients as compared to the healthy controls are provided in Fig. 1.

**Table 1:** Patient demographics of the investigated Alzheimer’s disease subtypes

	aAD (*n* = 19)	PCA (*n* = 7)	lvPPA (*n* = 6)	*p*-value
Age (years)	72 ± 9	65 ± 11	70 ± 8	0.346
Sex (F:M)	13:6	3:4	1:5	0.122
MMSE score (points)	22 ± 6	18 ± 10	13 ± 10	0.113

*aAD* amnestic Alzheimer’s disease; *F* female; *M* male; *MMSE* mini mental state examination; *lvPPA* logopenic variant primary progressive aphasia; *PCA* posterior cortical atrophy

### Tau accumulation patterns differ between aAD, lvPPA and PCA patients

**Fig. 2:**
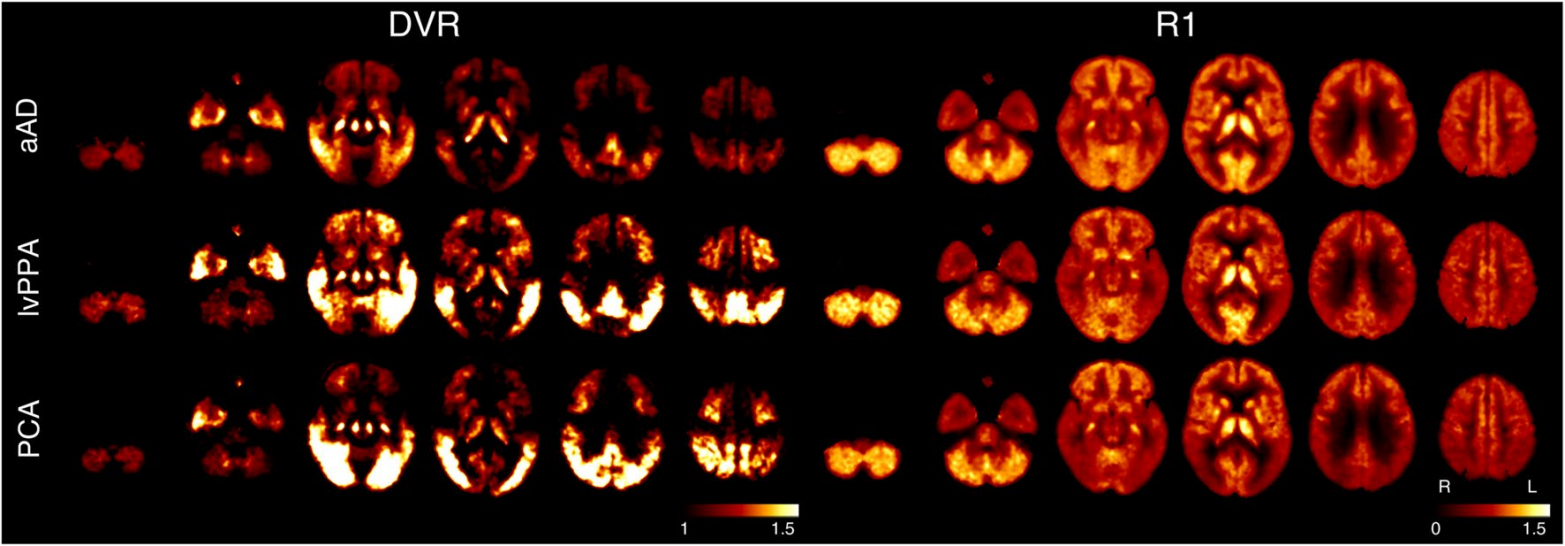
[^18^F]PI-2620 DVR and R1 maps in Alzheimer’s disease subtypes. aAD: amnestic Alzheimer’s disease; DVR: distribution volume ratio; L: left; lvPPA: logopenic variant primary progressive aphasia; PCA: posterior cortical atrophy; R: right; R1: relative perfusion

**Fig. 3:**
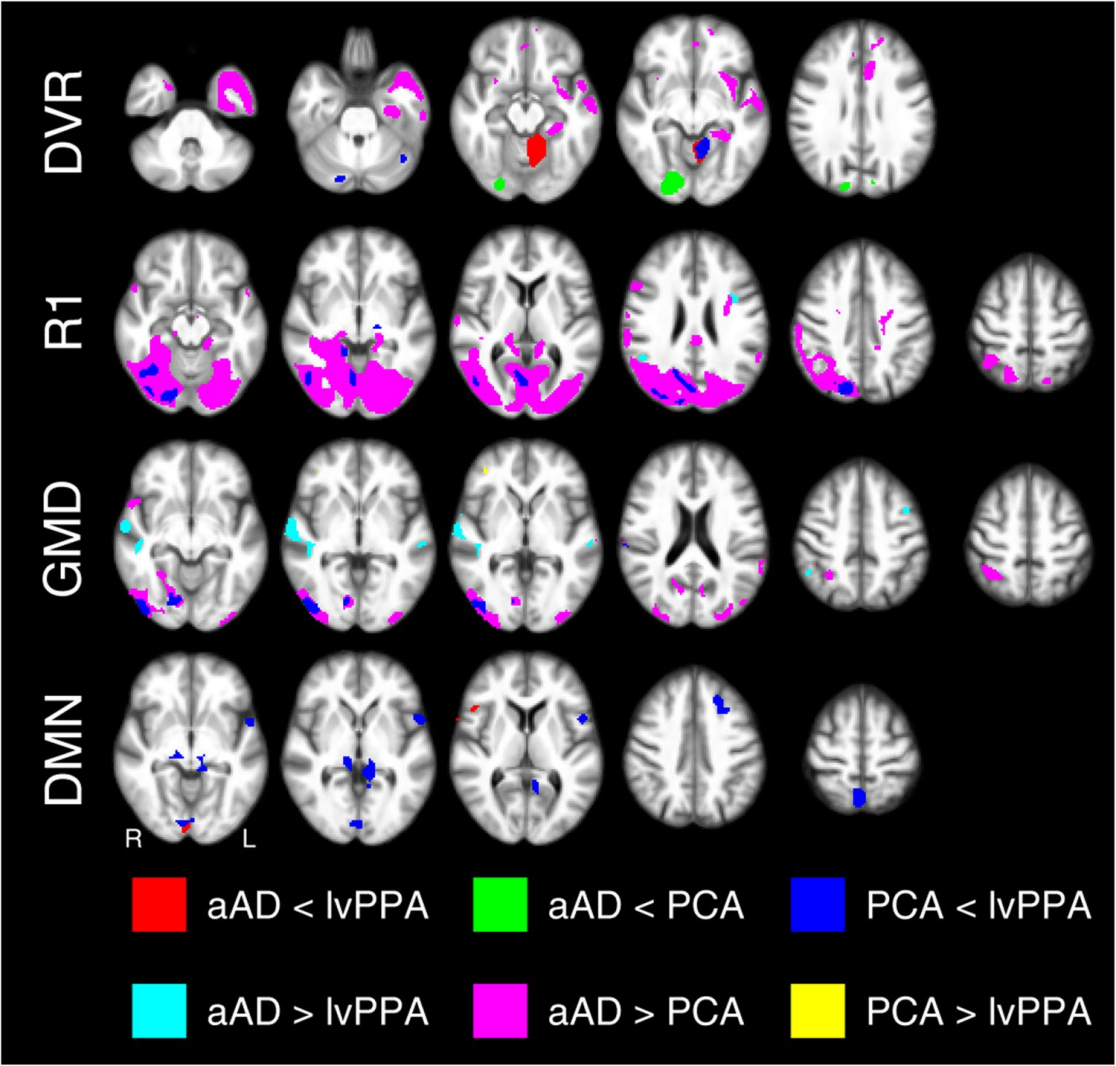
Voxel-based multi-parametric [^18^F]PI-2620 PET/MR image differences between Alzheimer’s disease subtypes. aAD: amnestic Alzheimer’s disease; DMN: default mode network; DVR: distribution volume ratio; GMD: grey matter density; L: left; lvPPA: logopenic variant primary progressive aphasia; PCA: posterior cortical atrophy; R: right; R1: relative perfusion

Analysis of the [^18^F]PI-2620 PET images revealed that aAD patients showed significantly higher DVRs in the temporal lobe, anterior cingulate cortex and anterior prefrontal cortex compared to PCA patients. In turn, patients suffering from PCA showed significantly higher DVRs in secondary visual cortex and visual associative cortex as compared to aAD patients. Furthermore, lvPPA patients revealed significantly higher DVRs in the vermis of the cerebellum as compared to aAD patients. When compared to PCA patients, significantly higher DVRs were observed in the cerebellar vermis and cerebellar declive. Figure 2 provides an overview of regions of tau accumulation in PCA and lvPPA patients and Fig. 3 shows which areas can be used to distinguish the here described different AD phenotypes. Table 2 provides a more detailed overview of the aforementioned changes.

**Table 2:** MNI coordinates and corresponding brain areas and Brodmann areas for the voxel-based [^18^F]PI-2620 DVR differences between Alzheimer’s disease subtypes

*Brain regions that show higher DVRs in aAD patients as compared to PCA patients*				
x [mm]	y [mm]	z [mm]	Area	Brodmann area
−32	−10	−42	Left Inferior Temporal Gyrus	20
20	8	−36	Right Temporal Pole	38
−6	26	26	Left Dorsal Anterior Cingulate Cortex	32
−16	54	24	Left Anterior Prefrontal Cortex	10
*Brain regions that show higher DVRs in PCA patients as compared to aAD patients*				
x [mm]	y [mm]	z [mm]	Area	Brodmann area
22	−88	−10	Right Secondary Visual Cortex	18
16	−88	30	Right Visual Associative Cortex	19
*Brain regions that show higher DVRs in lvPPA patients as compared to aAD patients*				
x [mm]	y [mm]	z [mm]	Area	Brodmann area
−10	−58	−14	Left Cerebellar Vermis	N/A
*Brain regions that show higher DVRs in lvPPA patients as compared to PCA patients*				
x [mm]	y [mm]	z [mm]	Area	Brodmann area
16	−80	−28	Right Cerebellar Declive	N/A
−12	−48	−8	Left Cerebellar Vermis	N/A

*aAD* amnestic Alzheimer’s disease; *DVR* distribution volume ratio; *lvPPA* logopenic variant primary progressive aphasia; *PCA* posterior cortical atrophy

### Hypoperfusion and grey matter density decrease shows the origin of cortical deficits in PCA and lvPPA and may help to discern aAD patients from other phenotypes

R1s were lower in the occipital lobe (*i.e.*, the visual motor cortex and the visual associative cortex) in PCA patients as compared to aAD patients and lvPPA patients. VBM analysis showed decreased GMD of the identical regions of the occipital lobe in PCA patients as compared to lvPPA patients.

In lvPPA patients, R1s were lower in the inferior part of the left sided frontal operculum (Broca operculum) as compared to aAD patients. VBM analysis, on the other hand, showed significantly decreased GMD in the left medial temporal gyrus, left premotor and supplementary motor areas, the left angular gyrus and the right superior temporal gyruss and right supramarginal gyrus in lvPPA patients as compared to aAD patients. Figure 3, Tables 3 and 4 provide a more detailed overview of the aforementioned changes.

**Table 3:** MNI coordinates and corresponding brain areas and Brodmann areas for the voxel-based [^18^F]PI-2620 R1 differences between Alzheimer’s disease subtypes

*Brain regions that show lower R1 in PCA patients as compared to lvPPA patients*				
x [mm]	y [mm]	z [mm]	Area	Brodmann area
14	−76	42	Right Visual Motor Cortex	7
12	−44	−4	Right Visual Associative Cortex	19
*Brain regions that show lower R1 in lvPPA patients as compared to aAD patients*				
x [mm]	y [mm]	z [mm]	Area	Brodmann area
−36	6	26	Left Broca Operculum	44

*aAD* amnestic Alzheimer’s disease; *lvPPA* logopenic variant primary progressive aphasia; *PCA* posterior cortical atrophy; *R1*: relative perfusion

**Table 4:** Results of VBM analyses displayed as MNI coordinates and corresponding brain areas and Brodmann areas

*Loss of GMD in brain regions of aAD patients as compared to PCA patients*				
x [mm]	y [mm]	z [mm]	Area	Brodmann area
33	−88.5	0	Right Secondary Visual Cortex	18
30	−51	55.5	Right Visual Motor Cortex	7
−24	−93	12	Left Secondary Visual Cortex	18
55.5	16.5	−7.5	Right Broca Operculum Region	44
−7.5	−66	19.5	Left Primary Visual Cortex	17
43.5	−37.5	−28.5	Left Inferior Temporal Gyrus	20
−64.5	−49.5	16.5	Left Angular Gyrus	39
−15	−84	45	Left Visual Associative Cortex	19
−60	−52.5	31.5	Left Angular Gyrus	39
63	−19.5	15	Right Supramarginal Gyrus	40
*Loss of GMD in brain regions of PCA patients as compared to lvPPA patients*				
49.5	−81	−6	Right Visual Associative Cortex	19
18	−78	−6	Right Secondary Visual Cortex	18
*Loss of GMD in brain regions of lvPPA patients as compared to aAD patients*				
63	−4.5	−6	Right Superior Temporal Gyrus	22
−42	7.5	49.5	Left Premotor + Supplementary Motor Area	6
−61.5	−52.5	31.5	Left Angular Gyrus	39
52.5	−39	−4.5	Left Medial Temporal Gyrus	21
49.5	−51	49.5	Right Supramarginal Gyrus	40
−60	−22.5	−1.5	Left Superior Temporal Gyrus	22

*aAD* amnestic Alzheimer’s disease; *GMD* grey matter density; *lvPPA* logopenic variant primary progressive aphasia; *PCA* posterior cortical atrophy

### Loss of functional connectivity of the default mode network in aAD and other AD phenotypes

In PCA patients, functional connectivity of the PCC was reduced with, among other brain regions, the visual motor area, parahippocampal gyrus, primary visual cortex and Broca operculum as compared to lvPPA patients (Table 5).

**Table 5:** Results of functional connectivity analyses displayed as MNI coordinates and corresponding brain areas and Brodmann areas

*Brain regions that show a loss of functional connectivity with the posterior cingulate gyrus in PCA patients as compared with lvPPA patients*				
x [mm]	y [mm]	z [mm]	Area	Brodmann area
5	−57	63	Right Visual Motor Area	7
−10	−39	0	Left Parahippocampal gyrus	36
−10	−51	6	Left Ventral Posterior Cingulum	23
−10	−24	−6	Dorsal Mesencephalon Left	N/A
−22	30	45	Left Frontal Eye Fields	8
11	−24	−6	Dorsal Mesencephalon Right	N/A
8	−30	0	Right Thalamus	N/A
8	−87	−3	Right Primary Visual Cortex	17
−55	12	0	Left Broca Operculum	44
*Brain regions that show a loss of functional connectivity with the posterior cingulate gyrus in aAD patients as compared with lvPPA patients*				
x [mm]	y [mm]	z [mm]	Area	Brodmann area
44	24	12	Right Broca Triangle	45
62	15	15	Right Broca Operculum	44
8	−84	−18	Right Cerebellar Declive	N/A
5	−90	−6	Right Secondary Visual Cortex	18

*aAD* amnestic Alzheimer’s disease; *lvPPA* logopenic variant primary progressive aphasia; *PCA* posterior cortical atrophy

Patients with aAD showed reduced functional connectivity of the PCC with the Broca triangle, Broca operculum, cerebellar declive and secondary visual cortex as compared to lvPPA patients. Figure 3 provides an overview of brain regions with distinctly different patterns in activity of the default mode network between aAD patients and PCA and lvPPA patients.

### Deteriorated microstructural organization of the (left) cingulum in lvPPA patients

In the lvPPA patients, the bilateral cingulum had lower FA values as compared to the aAD patients (F = 4.154; *p* = 0.029). This effect was primarily caused by lower FA values of the left cingulum in the lvPPA patients (F = 4.792; *p* = 0.029). The FA value of the right cingulum, on the other hand, was not significantly different between lvPPA or aAD patients (F = 2.537; *p* = 0.098). FA values of the cingulum on either side or bilaterally were not significantly different when comparing other subgroups included in this study. One-way ANOVA showed no other significant differences in FA values of the other white matter tracts (i.e., fornix, arcuate fasciculus, optic radiation) on either side or bilaterally between subgroups.

## Discussion

In this hybrid PET/MRI study with the second-generation tau PET tracer [^18^F]PI-2620, we provide evidence that phenotyping AD patients (i.e., aAD, lvPPA, PCA) is possible with regard to tau accumulation, relative brain perfusion, grey matter density, functional network alterations and microstructural white matter alterations. Especially with regard to the described differences in regional tau binding between lvPPA and PCA patients as compared to amnestic AD patients, we underline the importance of refinements of the Braak staging system for these AD subtypes as suggested by Macedo et al. [20]. Nevertheless, histopathological studies of AD phenotypes suggested that, despite regional differences in neurofibrillary tangle densities which characterize different focal cortical syndromes, the Braak staging model can still be meaningfully applied [32, 33].

The occipital lobe, the primary visual cortex in particular, was found to undergo changes in relative perfusion, tau accumulation and functional connectivity in PCA patients, which corresponds to available evidence on the pathophysiology of PCA. Similar findings of co-occurrence of tau accumulation in the primary visual cortex and functional connectivity disturbances have been described recently by Sintini et al. [34]. Although the present study cannot provide insights in amyloid accumulation in these brain regions, the current results are in line with the cascading network failure theory [35, 36]. In this theory, it is hypothesized that tau-associated local network failure is followed by a global compensatory phenomenon (which is associated with Aβ build up). However, when highly connected brain regions which integrate multi-source information—known as functional hubs (*e.g*. the primary visual cortex)—reach their limit of offering resilience to local network failures, tau accumulation within those “failing networks” accelerates rapidly. For that reason, the cascading network failure theory states that Aβ deposition is irrespective from the clinical subtype, whereas tau accumulation will vary by clinical phenotype.

The described findings of this [^18^F]PI-2620 PET/MRI study underpin the pathophysiological changes that occur in lvPPA patients in regions involved in language processing. Tau accumulation in the cerebellar vermis was significantly higher in lvPPA patients when compared to amnestic AD patients and PCA patients. The role of the cerebellum in language processing and production is well-known [37], although the function of the vermis is not well understood. Nevertheless, surgical incision of the vermis has been shown to represent a risk factor of developing post-operative language deficits (i.e., cerebellar mutism) [38, 39]. Furthermore, atrophy of the vermis was found to be correlated with language impairment in children [40]. Taken together, we suggest that the aforementioned tau deposits in the cerebellar vermis can be seen as a confirmation of the cascading network failure theory [35, 36]. The spatial distribution presented in this study seems to contradict the cortical tau maps presented by others, which show left greater than right tau pathology in lateral temporal, lateral parietal, precuneus, and posterior cingulate cortices [41, 42]. However, significant overlap between cortical tau maps of lvPPA and PCA [41] and lvPPA and amnestic AD patients [43] has been described as well, explaining why the we only found specific tau accumulation in the cerebellar vermis when comparing these disorders.

Relative hypoperfusion of the left frontal operculum was also observed in the present study for the lvPPA patients. This brain region, also known as Broca’s area, is prominently involved in language production and is a well-known affected region in non-fluent variant PPA [44]. However, not specific on relative hypoperfusion, previous studies reported on neurodegeneration and tau accumulation in the left frontal operculum in lvPPA patients as well [41, 42]. Also, we found decreased GMD in the right superior temporal gyrus in lvPPA patients, which is part of the primary auditory cortex. However, the superior temporal gyrus, in both hemispheres, is also a central hub within the semantic cognition network and, as such, involved in the production and understanding of language which also supports many non-verbal behaviors [45, 46]. Finally, region of interest analysis of white matter bundles involved in cognition, processing language and conduction of visual input showed that microstructural integrity of the (left-sided) cingulum was significantly lower in lvPPA patients as compared to amnestic AD patients. These results are in keeping with data of other groups showing a relevant role of the cingulum in language processing [47, 48].

This study shows that a single visit multi-parametric [^18^F]PI-2620 tau PET/MRI provides a wealth of functional and structural data which can be used to discern aAD patients from other AD phenotypes. The prospective inclusion of patients and the multiparametric imaging protocol are regarded as two strengths. Nonetheless, the current study also inherently suffers from some limitations. The limited sample size of included variants of AD patients affects the study’s power and might affect the generalizability to larger cohorts. Another limitation concerns the restricted demographic information provided, especially with regard to the clinical status of cognition, vision, language production and language comprehension. Furthermore, one recent report advocated against the use of the cerebellar cortex as a reference region in studies using [^18^F]PI-2620 since the dentate nucleus is an on-target binding site of this second-generation tau PET tracer [49]. Furthermore, here presented results also reveal tau accumulation in the cerebellum in PCA and lvPPA, indicating that the cerebellum might not be a suitable reference region. Although, the inferior cerebellum was taken as a reference region in this study, de facto excluding the dentate nucleus and other cerebellar structures that showed tau accumulation. However, it remains difficult to oversee to what extent this chosen reference region affected the outcomes as compared to when the fusiform gyrus would have been used as a reference region, as recently proposed Bischof et al. [49]. Regarding the here described subtle differences in tau accumulation patterns between aAD, lvPPA and PCA patients, we stress the importance for future studies which should aim to provide tau accumulation profiles in larger cohorts of lvPPA and PCA patients.

## Conclusion

This hybrid [^18^F]PI-2620 PET/MRI study provides evidence that phenotyping of aAD, lvPPA and PCA patients is possible with regard to tau accumulation, relative brain perfusion, grey matter density, functional network alterations and microstructural white matter alterations. Especially with regard to the described differences of the regional tau accumulation between aAD, lvPPA and PCA patients, we underline the importance of refinements in the Braak staging system. Furthermore, these patterns of tau accumulation align with the cascading network failure hypothesis. More research is needed to reproduce the here presented results in larger patient cohorts.

## Data Availability

The datasets generated and analysed during the current study are available from the corresponding author on reasonable request. The data are not publicly available due to their containing information that could compromise the privacy of the participants.
